# Caveat when using ADC(2) for studying the photochemistry of carbonyl-containing molecules†

**DOI:** 10.1039/d1cp02185k

**Published:** 2021-06-04

**Authors:** Emanuele Marsili, Antonio Prlj, Basile F. E. Curchod

**Affiliations:** Department of Chemistry, Durham University Durham DH1 3LE UK antonio.prlj@durham.ac.uk basile.f.curchod@durham.ac.uk

## Abstract

Several electronic-structure methods are available to study the photochemistry and photophysics of organic molecules. Among them, ADC(2) stands as a sweet spot between computational efficiency and accuracy. As a result, ADC(2) has recently seen its number of applications booming, in particular to unravel the deactivation pathways and photodynamics of organic molecules. Despite this growing success, we demonstrate here that care has to be taken when studying the nonradiative pathways of carbonyl-containing molecules, as ADC(2) appears to suffer from a systematic flaw.

## Abstract 2

We highlight a shortcoming of the (SCS)-ADC(2) method when studying the photophysics and photochemistry of carbonyl-containing molecules.

## Graphical abstract

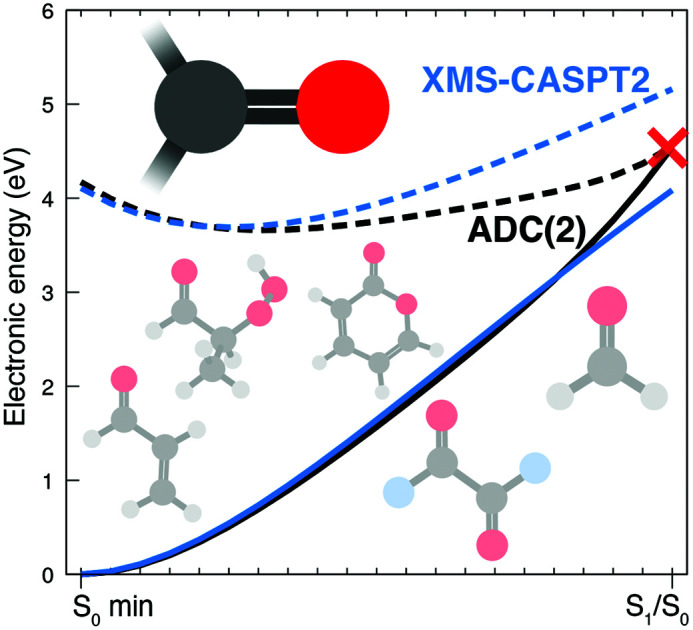

The theoretical description of photophysics and photochemistry of molecules and materials has matured enough that nowadays most experimental observations can be successfully interpreted and sometimes predicted fully *in silico*. *Ab initio* calculations have contributed substantially to understanding the molecular mechanisms behind the human and animal vision,^1^ optimizing solar cells^2^ and light-emitting devices,^3^ designing molecular motors and rotors,^4^ dyes^5^ or photochemical switches,^6^ understanding complex photobiological,^7^ atmospheric^8^ or interstellar^9^ phenomena, *etc.* The processes underlying all these applications involve the interplay and interconversion between ground and excited electronic states.

Molecular electronic states are rigorously obtained as the solutions of the time-independent electronic Schrödinger equation. While an exact solution of this Schrödinger equation is an unattainable goal for nearly all molecular systems, a number of approximate electronic-structure methods has been developed and used to study realistic molecular systems. The ground state of a standard closed-shell organic molecule is typically well described by a single closed shell electronic configuration, using popular electronic-structure methods like Kohn-Sham density-functional theory (DFT) or Møller-Plesset perturbation theory up to second-order (MP2). In more challenging situations, one needs to resort to so-called multireference approaches, where the ground-state wavefunction is described by a linear combination of multiple electronic configurations with sizable weights. Along with the ground state, excited states can also be obtained with methods like extended multi-state complete active space second-order perturbation theory (XMS-CASPT2) and multi-reference configuration interaction singles and doubles (MR-CISD).^10,11^ Such multireference methods often require the careful selection of key orbitals forming an active space, and are truly applicable only to relatively small molecular systems due to their computational burden. However, they allow for an accurate description of conical intersections (CIs) and their branching space–key ingredients for the understanding of nonradiative processes.

Substantially cheaper than multireference methods, strategies based on a single reference or on DFT have gained a large popularity in the computations of excited states. Linear-response time-dependent density-functional theory (TDDFT), an extension of DFT to excited states, is perhaps the most popular method for excited states nowadays. TDDFT (and its Tamm-Dancoff approximation, TDA) has become a convenient tool to support, predict, or interpret the experimental data in molecular spectroscopy,^12^ and can be combined with excited-state molecular dynamics simulations.^13^ Over the years, different failures of standard TDDFT (and underlying DFT) approximations have been documented for certain types of excited states (*e.g.* charge-transfer states, doubly-excited states), meaning that the use of TDDFT requires careful benchmarks with respect to higher levels of theory.^14^ Importantly, gaining knowledge in the failures of TDDFT approximations has also contributed to a better and safe use of this efficient electronic-structure method.

A commonly employed single-reference method is the algebraic diagrammatic construction of second order, ADC(2), an extension of MP2 to excited states.^15^ ADC(2) is not as computationally affordable as TDDFT, but also not as computationally cumbersome as multireference approaches. It shares the black-box nature of TDDFT, which makes it an appealing approach for wide applications in spectroscopy and molecular dynamics. Just like in TDDFT, the accuracy of ADC(2) critically depends on the quality of the ground-state reference–situations where MP2 fails to describe the ground electronic state are detrimental for the description of the excited states. Nevertheless, ADC(2) appears to be a more reliable method than TDDFT, granting a balanced description of excited-state energies for standard organic molecules, with a relatively low mean error around 0.2 eV.^15^ While the development of ADC(2) method dates back to 1982,^16^ its wide applications to photochemistry and photophysics are not older than a decade. Among relevant studies, Tuna *et al.*^17^ showed that ADC(2) does not properly describe the topology of conical intersections between the ground (S_0_) and the first excited (S_1_) singlet state (while multireference methods do), yet it can still predict reasonable crossing energies and geometries. Plasser *et al.*^18^ investigated the application of different single-reference methods in excited-state molecular dynamics of adenine, placing ADC(2) as a serious competitor to the commonly-accepted TDDFT for nonadiabatic dynamics. Following these reports, an explosion of studies employing ADC(2) in excited-state dynamics appeared in the literature,^19–33^ also mapping the reaction paths between the Franck-Condon (FC) geometry and the electronic states crossings,^28–45^ and calculating absorption properties of functional molecules.^46,47^ The community has gained a large confidence with ADC(2), to the point where many studies employing it do not contain systematic comparisons with high-level multireference methods.^19,24,26–29,38–42^ This is understandable though considering that the latter studies involve middle-sized or bigger molecular systems. For these systems ADC(2) is an ace in the hole, appearing as an ideal compromise between accuracy and computational efficiency.

In this communication, we highlight what appears to be a systematic issue of ADC(2) in describing some electronic states of molecule bearing a carbonyl group, which concerns a large class of molecules like chromophores, nucleobases, or atmospheric volatile organic compounds. Such systems possess characteristic low-lying singlet excited state of *n*(O)π*(C=O) character (shortened by *n*π* in the following), where *n*(O) is the lone pair orbital associated to the oxygen of the carbonyl group and π*(C=O) refers here to an unoccupied π-type orbital localized either fully on the carbonyl functional group or partially when delocalization is possible. We start our investigation by examining the ADC(2) excited-state dynamics of small molecules bearing carbonyl moiety. Fig. 1 shows the time evolution of the ground (solid lines) and excited (dashed lines) state electronic energies following the molecular dynamics initiated by a *n*π* photoexcitation. The electronic states were computed with the spin-component-scaled (SCS) variant of MP2/ADC(2), which was recently shown to improve the potential energy surfaces,^48^ combined with a def2-SVP basis set (abbreviated SVP in the following, see ESI† for the full computational details and a comparison between SCS-ADC(2) and ADC(2)–in the following we will note SCS-MP2/ADC(2) to denote the combination of SCS-MP2 for S_0_ and SCS-ADC(2) for the excited states). Inspecting the time trace of the electronic energies for five different carbonyl-bearing molecules, namely formaldehyde, (anti-)acrolein, pyrone, 2-hydroperoxy-propanal (2-HPP), and oxalyl fluoride, reveals a common feature: all the systems appear to possess an easily accessible nonradiative decay channel *via* a S_1_/S_0_ crossing, achieved by an ultrafast elongation of the C=O bond. Interestingly, a similar nonradiative pathway showed up in the excited-state dynamics of the nucleobase thymine, accounting for 61% of radiationless decay in gas phase,^49^ and 54% in water.^26^ The decay channel associated with C=O elongation was also identified in a guanine derivative,^32^ though it was argued that other deactivation mechanisms would prevail. Interestingly, all these studies employed ADC(2) method. As such, a nonradiative decay channel mediated by the C=O elongation appears to be a general and well-established feature in excited-state dynamics of carbonyl-containing molecules. Can it really be the case? In fact, more systematic studies of cytosine^33,50^ and guanine^31^ derivatives photophysics cast some shadow of doubt on these observations. In these studies, ADC(2) predictions were compared to the results from a high-level multireference method, finding some conspicuous discrepancies. What does that mean for the molecules shown in Fig. 1? We will show in the following that such an easily-accessible deactivation pathway is an artefact of the MP2/ADC(2) method that end users should be aware of.

**Fig. 1:**
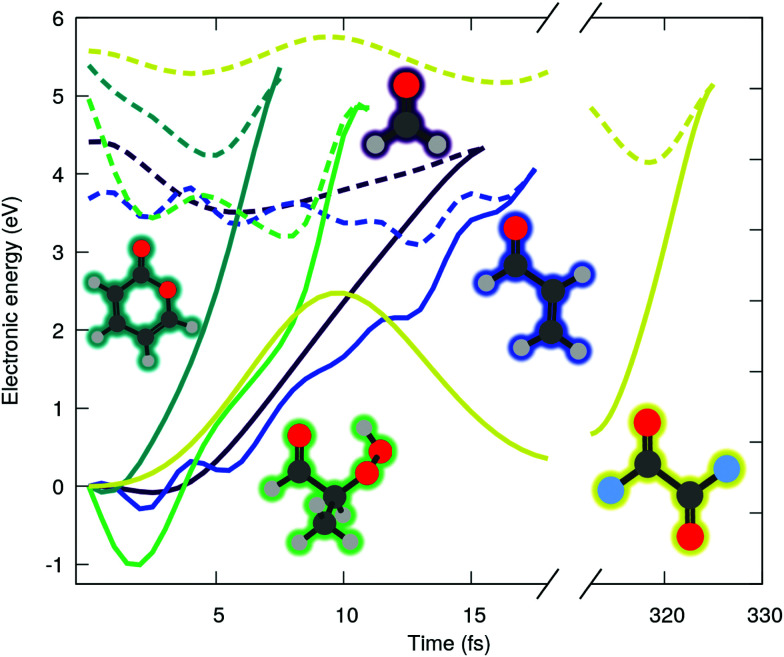
Time evolution of the electronic energies of the running excited (dashed line) and S_0_ (solid line) states of formaldehyde (purple), acrolein (blue), pyrone (dark green), 2-HPP (light green) and oxalyl fluoride (yellow), following an excited-state molecular dynamics initiated in an *n*π* electronic state (SCS-MP2/ADC(2)).

To highlight the artificial nature of these nonradiative pathways, we optimized the S_0_ minimum geometry–the Franck-Condon (FC) point–with SCS-MP2/SVP and the S_1_/S_0_ crossing point (S_1_/S_0_ CP) with SCS-MP2/ADC(2)/SVP. We then interpolated geometries in internal coordinates between these two critical points and computed electronic energies both with SCS-MP2/ADC(2) and XMS-CASPT2/SVP (see ESI† for additional details on these calculations, active space, SCS-ADC(2) *vs.* ADC(2), and influence of the basis set). Surprisingly, XMS-CASPT2 shows an entirely different behavior when the C=O bond is extended beyond 1.4 Å. The intersection observed between S_0_ and S_1_ (*n*π*) at the SCS-MP2/ADC(2) level is not present at the XMS-CASPT2 for all molecules. Any attempt to locate the crossing at XMS-CASPT2 level did not lead to a structure resembling the S_1_/S_0_ CP of SCS-MP2/ADC(2). In other words, SCS-MP2/ADC(2) suggests the presence of an easily accessible S_1_/S_0_ CP, while XMS-CASPT2 strongly indicates that the crossing is not easily accessible–whether it is much higher in energy or does not exist at all. Importantly, such a discrepancy between the two methods is systematic for all five molecules. We also note that ADC(2) shows the same artificial crossings (see ESI†).

Let us now focus on the case of formaldehyde to gain deeper insights in this issue. Fig. 3 shows the same electronic-energy profiles as in Fig. 2 but now for five different methods: TDA-PBE0/SVP (grey), SCS-ADC(2)/SVP (black), XMS(2)-CASPT2(2/2)/SVP (red), XMS(2)-CASPT2(4/3)/SVP (blue) and MR-CISD(4/3)/SVP (green). For details about these methods see ESI.† The high-level methods XMS(2)-CASPT2(4/3) and MR-CISD both agree on the absence of an intersection at the SCS-MP2/ADC(2) S_1_/S_0_ CP geometry. The D_1_ diagnostic (dashed orange) measures the degree of multiconfigurational character for the MP2 ground state. The quick D_1_ surge beyond the recommended limit value of 0.04^51^ (and even the less conservative value of 0.1 proposed by others^52^) indicates that the S_0_ state acquires a multiconfigurational character along the pathway. In other words, the single-reference wavefunction that serves as reference for perturbation theory is no more adequate for large D_1_ values. Interestingly, the PBE0 ground state appears to have an incorrect shape in comparison to high-level methods, but as the S_1_ electronic energy is also raising steeply along the profile, the S_1_/S_0_ CP is (fortuitously) avoided. The artificial S_1_/S_0_ CP of SCS-MP2/ADC(2) can be explained by the combination of two recurring factors for all molecules bearing a carbonyl group: (i) the MP2 ground state is not adequate and overestimates the S_0_ energy when stretching the C=O bond and (ii) the curvature of the *n*π* state along the CO stretching coordinate is too small. The poor description of S_1_ can be correlated with an increasing contribution of doubly-excited configurations along the interpolation pathway (see Fig. S2 in the ESI†). Such contributions cannot be optimally described by ADC(2) since the treatment of double excitations in ADC(2) is very limited.^15^ Finally, the point (ii) resonates with earlier findings that ADC(2) was inaccurate in describing the *n*π* state of cytosine,^48^ (even if SCS-ADC(2) was shown to perform better), while it has been pointed out that ADC(2) performs less accurately when describing *n*π* transition, yielding too low frequencies associated with the carbonyl stretching.^53^ Another study also showed that ADC(2) already overestimates the C=O bond length of formaldehyde at its S_1_ minimum.^54^

**Fig. 2:**
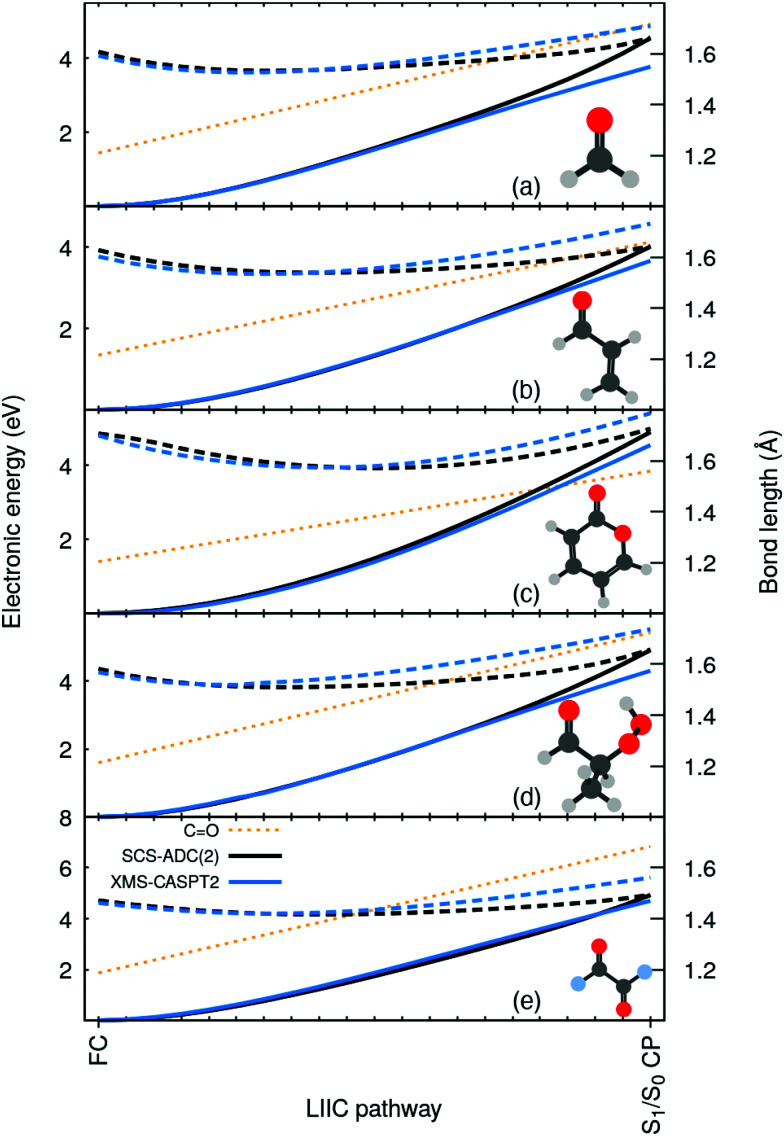
Electronic energies along a linear interpolation in internal coordinates (LIIC) between the FC and the S_1_/S_0_ CP for: (a) formaldehyde, (b) acrolein, (c) pyrone, (d) 2-HPP, and (e) oxalyl fluoride, as obtained with SCS-MP2/ADC(2)/SVP (black) and XMS-CASPT2/SVP (blue). A solid (dashed) line is used for S_0_ (S_1_), and a dotted orange line for the C=O bond length.

Based on the considerations above, we could reproduce the S_1_/S_0_ CP using XMS-CASPT2 by employing a minimal active space constituted only by two electrons in *n*(O) and the π*(C=O) (XMS(2)-CASPT2(2/2) in Fig. 3). Such a small active space does not include the π orbital, which is a key contributor to the multiconfigurational character of the S_0_ along the pathway, when the closed-shell configuration starts to strongly mix with a ππ* contribution (see Fig. S2 in the ESI†). As a result, XMS(2)-CASPT2(2/2) leads, as for MP2, to a poor description of the ground-state reference wavefunction on which perturbation theory is applied, ultimately leading to a failure of the method when reaching the S_1_/S_0_ CP region (in stark contrast with XMS(2)-CASPT2(4/3) and MR-CISD, both including the π orbital). Therefore, the failure of XMS-CASPT2 caused by the small active space appears to mimic that of SCS-ADC(2). The very same trends are systematically reproduced for the other four compounds, as depicted in Fig. S6 in the ESI.† Hence, our observations point towards (i) a too shallow S_1_(*n*π*) along the elongation of the CO bond combined with (ii) a bad reference for MP2 destabilizing too rapidly the ground state to explain the fast and artificial decay observed in the excited-state dynamics. We note that the point (ii) resonates with earlier findings that ADC(2) tends to have energy gaps between S_1_ and S_0_ closing too rapidly even still far from the crossing region (*e.g.* ref. 55 and 56).

**Fig. 3:**
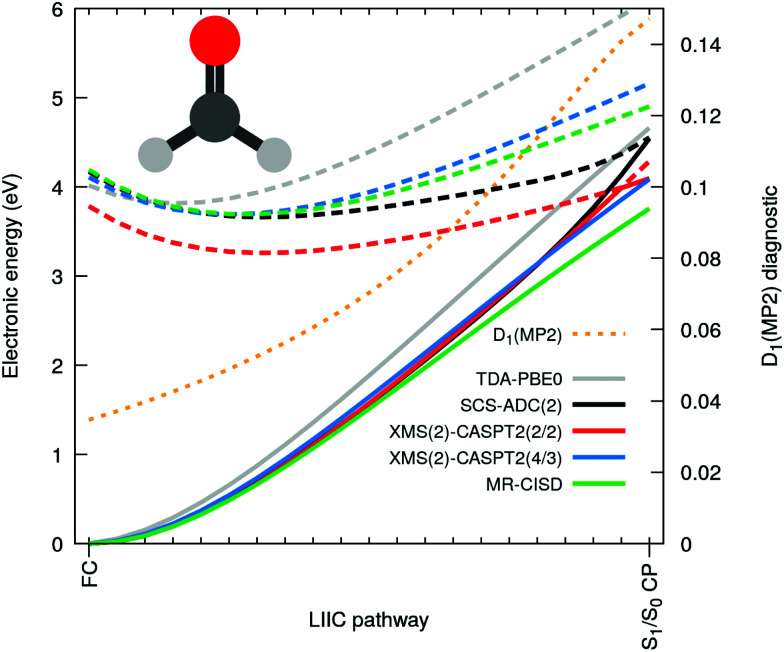
Electronic energies along a linear interpolation in internal coordinates between the FC and the S_1_/S_0_ CP for formaldehyde as obtained with SCS-ADC(2) (black), TDA-PBE0 (grey), XMS(2)-CASPT2(2/2) (red), XMS(2)-CASPT2(4/3) (blue) and MR-CISD (green). A solid (dashed) line is used for S_0_ (S_1_), and a dotted orange line for the D_1_ diagnostic.

We note that we also investigated the case of the aforementioned thymine nucleobase. Our rational explanation for the failure of both ADC(2) and XMS(2)-CASPT2(2/2) is clearly confirmed for this additional molecule: both ADC(2) and XMS(2)-CASPT2(2/2) predict an artificial S_1_/S_0_ crossing, which is verily avoided when using a suitably high level of theory (Fig. S7, ESI†).

In summary, the electronic-structure method ADC(2) is a rising star to study the photochemistry and photophysics of organic molecules, but its pitfalls still need to be fully uncovered. The unexpected failures of ADC(2) for carbonyl-containing compounds were discussed in several case studies but the systematic nature of these errors in the context of S_1_/S_0_ crossing points has not been widely recognized. The recurrence of artificial S_1_/S_0_ crossings upon C=O elongation closely resembles the predictions from multireference methods with an inadequate active space, which prevents the proper description of both ground and excited state, and leads to the unphysical nonradiative decay channels in molecular dynamics. Similar issues are expected for molecules bearing a C=S group, based on the results presented in ref. 57. Considering the omnipresence of C=O functional group and the increasing interest in using ADC(2) to study photochemical deactivation pathways, our current findings should serve as a warning bell for future research in the field.

## Conflicts of interest

There are no conflicts to declare.

## Supplementary Material

CP-023-D1CP02185K-s001

CP-023-D1CP02185K-s002

CP-023-D1CP02185K-s003

CP-023-D1CP02185K-s004

CP-023-D1CP02185K-s005

CP-023-D1CP02185K-s006

CP-023-D1CP02185K-s007
