# Supplementary material for: Caveat when using ADC(2) for studying the photochemistry of carbonyl-containing molecules
Source: Phys Chem Chem Phys. 2021 Jun 4;23(23):12945–9. doi: 10.1039/d1cp02185k (PMC8207513; doi:10.1039/d1cp02185k)
Supplement: CP-023-D1CP02185K-s001 [file CP-023-D1CP02185K-s001.pdf]

Electronic Supplementary Material (ESI) for Physical Chemistry Chemical Physics.
This journal is © the Owner Societies 2021

Electronic Supplementary Information for: Caveat when using ADC(2) for studying the photochemistry of carbonyl-containing molecules

Emanuele Marsili, Antonio Prlj,[a] and Basile F. E. Curchod[b]
Department of Chemistry, Durham University, South Road, Durham DH1 3LE, UK

CONTENTS

I. Computational Details 2
II. Additional information on formaldehyde 4
A. Contributing configurations along the LIIC 4
B. SCS-ADC(2) *vs* ADC(2), and the influence of the basis set 5
C. CC2 and SCS-CC2 *vs* SCS-ADC(2) 6
D. Influence of the vertical shift on XMS-CASPT2 profile 7
III. Comparing XMS-CASPT2(2/2) with XMS-CASPT2(4/3) for all molecules 8
IV. LIIC for thymine 10
References 11

[a] Electronic mail: antonio.prlj@durham.ac.uk
[b] Electronic mail: basile.f.curchod@durham.ac.uk

I. COMPUTATIONAL DETAILS

The following electronic-structure methods were used in this work: MP2 (Møller-Plesset perturbation theory up to second-order)[1]; (strict) ADC(2) (Algebraic Diagrammatic Construction up to second-order)[2–4] and its Spin-Component Scaled variant (SCS-ADC(2)), CC2 (Coupled-Cluster up to the second-order)[2,5] and SCS-CC2[6], DFT (Density Functional Theory)[7,8] and LR-TDDFT (Linear-Response Time-Dependent DFT)[9,10], SA-CASSCF (State-Averaged Complete Active Space Self-Consistent Field)[11], XMS-CASPT2 (extended Multi-State Complete Active Space second-order perturbation theory)[12,13] in its Single-State, Single-Reference (SS-SR, used if not stated otherwise) and Multi-State, Multi-Reference (MS-MR) versions[14,15], MR-CI (Multi Reference Configuration Interaction)[16] including single and double excitations MR-CISD. The calculations were performed with Gaussian09[17] (DFT and LR-TDDFT), Turbomole 7.3[18] (MP2, ADC(2), SCS-ADC(2), CC2 and SCS-CC2), BAGEL 1.2[19] (SA-CASSCF, SS-SR-XMS-CASPT2), and MOLPRO 2012.1[20,21] (SA-CASSCF, MS-MR-XMS-CASPT2, and MR-CISD) program packages.

The PBE0 exchange-correlation functional was used for all DFT and LR-TDDFT calculations[22,23], within the Tamm-Dancoff approximation. MP2, ADC(2), SCS-ADC(2), CC2, and SCS-CC2 were performed with frozen core and the Resolution of the Identity (RI)[24]. The standard scaling factors were used for SCS-ADC(2) and SCS-CC2. BAGEL calculations were performed using Density Fitting (DF) and frozen core. DF and frozen core approximations were not utilized in MOLPRO calculations. All XMS-CASPT2 calculations employed a real vertical shift of 0.5 Hartree unless otherwise stated (see Fig. S5 for a test of this value). The Karlsruhe basis sets def2-SVP and def2-TZVP were employed[25–27] – a def2-SVP basis set should be assumed if not stated otherwise.

For each molecule studied in this work, a linear interpolation in internal coordinates pathway was calculated between the Franck-Condon geometry (minimum on the S_0 potential energy surface, optimized at SCS-MP2/def2-SVP level of theory) and the S_1/S_0 crossing points (SCS-MP2/def2-SVP for S_0 and SCS-ADC(2)/def2-SVP for S_1). The minimum-energy crossing points close to the Franck-Condon region were located with CIOpt[28]. We note that while the CIOpt code in principle returns minimum-energy conical intersections, we prefer to coin the geometries obtained in this work 'crossing points' (CPs) given that ADC(2) does not describe adequately the branching space of S_0/S_1 conical intersections[29].

The standard notation XMS(k)-CASPT2(n/m) was used to indicate the details of the chosen active spaces and state-averaging protocol, with n electrons in m active orbitals while k states are considered. The reference XMS-CASPT2 energies for each molecule (using a SVP basis set) were obtained at the following level of theory: XMS(2)-CASPT2(6/5) for formaldehyde, XMS(2)-CASPT2(6/5) for acrolein, XMS(3)-CASPT2(6/5) for pyrone, XMS(4)-CASPT2(12/9) for 2-HPP, and XMS(3)-CASPT2(8/6)/def2-SVP for oxalyl-fluoride. The natural orbitals for these reference calculations are presented in Fig. S1. The reduced 4/3 active spaces were constructed by keeping only the n, π, and π^* orbitals localized on the carbonyl moiety (and a state-averaging over the two lowest electronic states).

Trajectory surface hopping dynamics employed Tully's fewest switches algorithm[30] and were performed with Newton-X version 2.0[31,32] using the Turbomole interface and the ADC(2) implementation discussed in Ref 33. The initial conditions were randomly selected

from a Wigner distribution for uncoupled harmonic oscillators, generated from a ground-state optimized geometry and corresponding vibrational frequencies obtained at SCS-MP2/def2-SVP level of theory. The excited-state dynamics were initiated in a $n\pi^*$ state for all molecules and employed a time step of 0.5 fs. Trajectories were stopped whenever they reach a region of configuration space where the S_1/S_0 energy gap was smaller than 0.01 eV. The default parameters of Newton-X were employed for all dynamics.

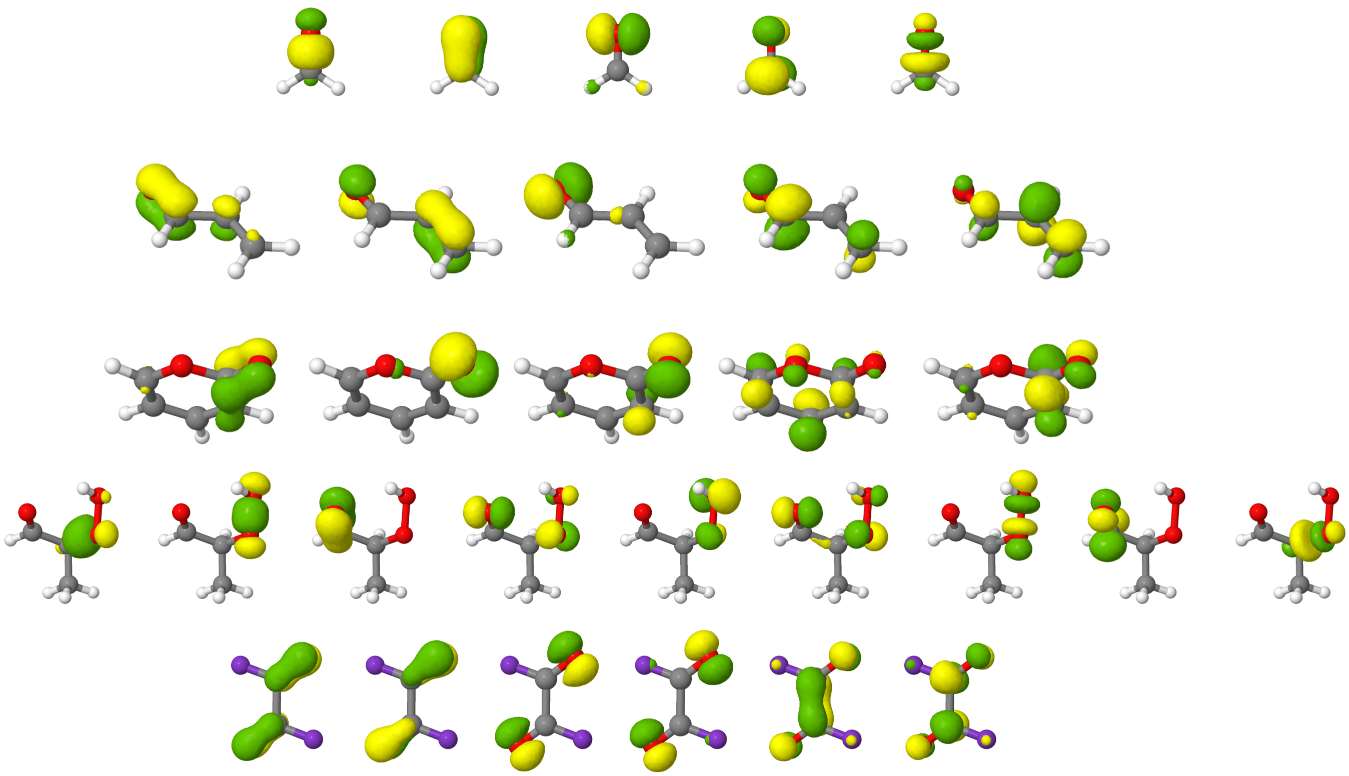

FIG. S1. SA-CASSCF natural orbitals (isovalue of 0.1) employed for the reference XMS-CASPT2 calculation (see text). Top to bottom: formaldehyde, acrolein, pyrone, 2-HPP, and oxalyl fluoride.

II. ADDITIONAL INFORMATION ON FORMALDEHYDE

A. Contributing configurations along the LIIC

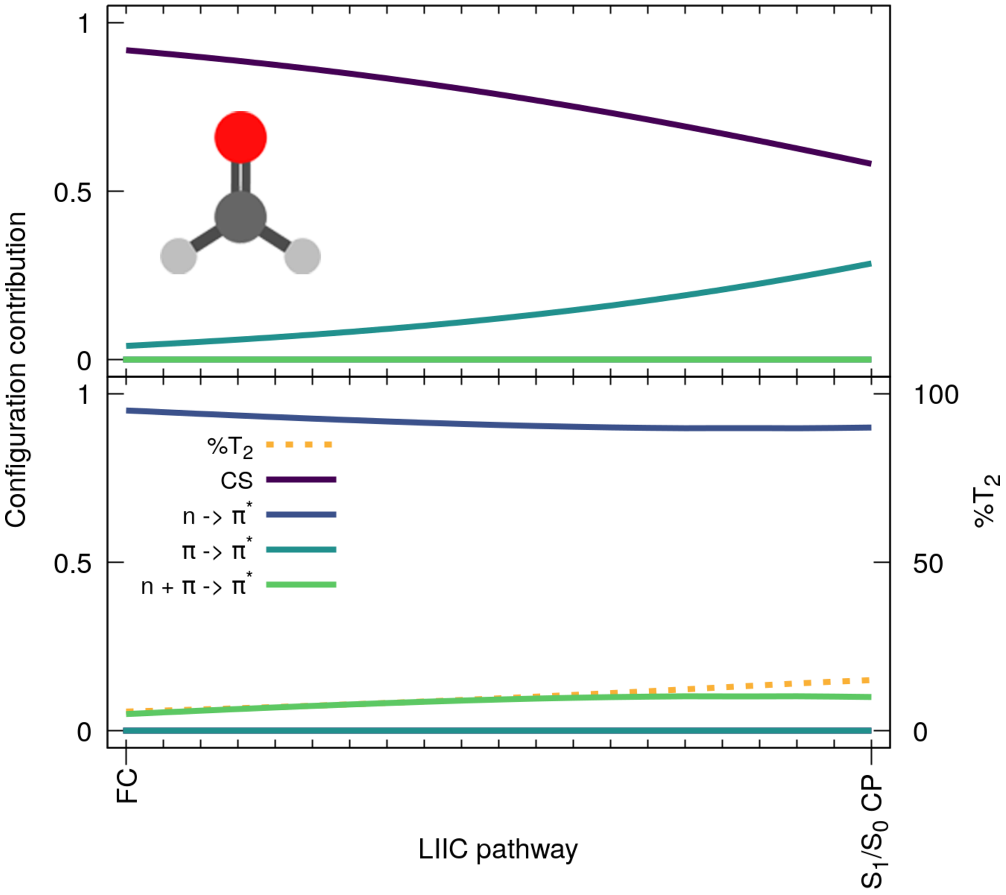

FIG. S2. Contributing configurations to the MS-MR-XMS(2)-CASPT2(4/3)/SVP wavefunctions along the formaldehyde LIIC pathway: closed shell (CS) configuration, singly-excited configurations $n\pi^*$ and $\pi\pi^*$, and doubly-excited configuration obtained from the promotion of a n and a π electron to the π^* orbital ($n + \pi \rightarrow \pi^*$). These configurations are plotted along the LIIC for the ground-state (upper panel) and first excited-state (lower panel) wavefunctions. The $\%T_2$, computed at the SCS-ADC(2)/SVP level of theory, is shown for S_1 with an orange dotted line.

Figure S2 shows the different contributions to the ground- and first excited-state wavefunctions based on MS-MR-XMS(2)-CASPT2(4/3), along the LIIC pathway of formaldehyde. Close to the FC region, the S_0 state is mostly characterized by a closed-shell (CS) configuration. Moving along the LIIC, one observes that the singly-excited configuration, $\pi\pi^*$, gains more importance. The increased weight of this configuration is in line with the dramatic increase of the D_1 diagnostic reported in the main text (Fig. 3). The S_1 state mostly preserves a dominant $n\pi^*$ configuration. We note a small increase in the doubly-excited configuration when progressing along the LIIC (in line with the increase in $\%T_2$ for SCS-ADC(2)).

B. SCS-ADC(2) vs ADC(2), and the influence of the basis set

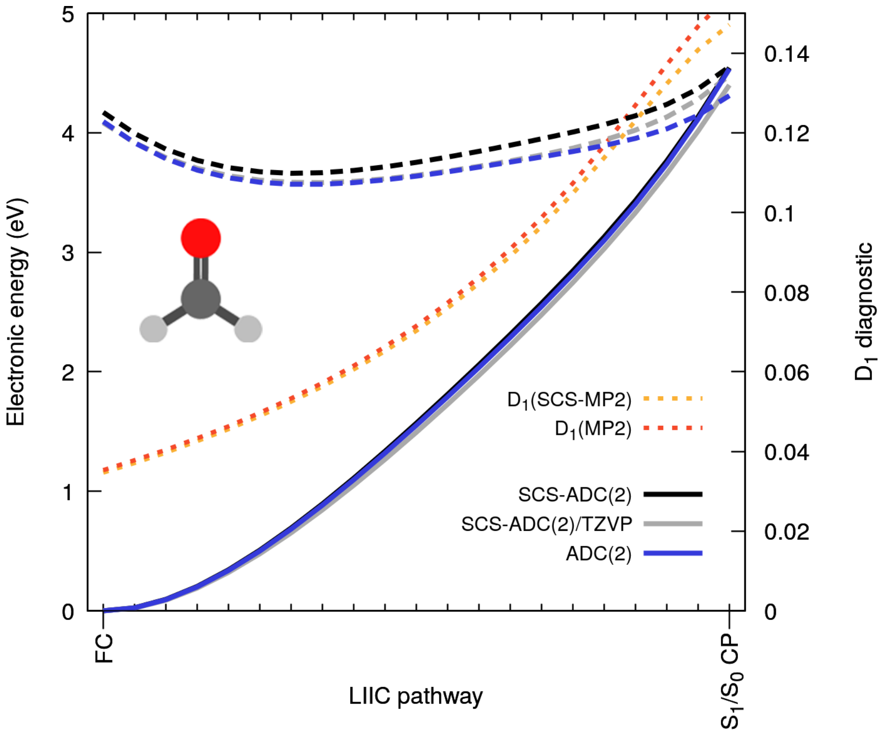

FIG. S3. Comparison between SCS-ADC(2)/SVP (black), SCS-ADC(2)/TZVP (grey) and ADC(2)/SVP (blue) for the LIIC of formaldehyde (solid line for S_0 and dashed line for S_1). The D_1 diagnostic is given by a dotted orange line (SCS-MP2) or a red line (MP2).

Figure S3 offers a confirmation that the spurious S_1/S_0 crossing observed in the main text is also present when employing a larger basis set (TZVP) or standard ADC(2).

C. CC2 and SCS-CC2 vs SCS-ADC(2)

Comparing the LIIC pathway for formaldehyde computed with CC2/SVP and SCS-CC2/SVP to the one obtained with SCS-ADC(2)/SVP (Fig. S4), it appears that CC2 and SCS-CC2 do not predict the same unphysical crossing between S_0 and $S_1(n\pi^*)$, despite a rather high D_1 diagnostic. This observation does not come as a complete surprise as numerous reports have shown that CC2 is capable, thanks to its formalism, of describing the branching space of S_1/S_0 rather accurately[29], in stark contrast with ADC(2).

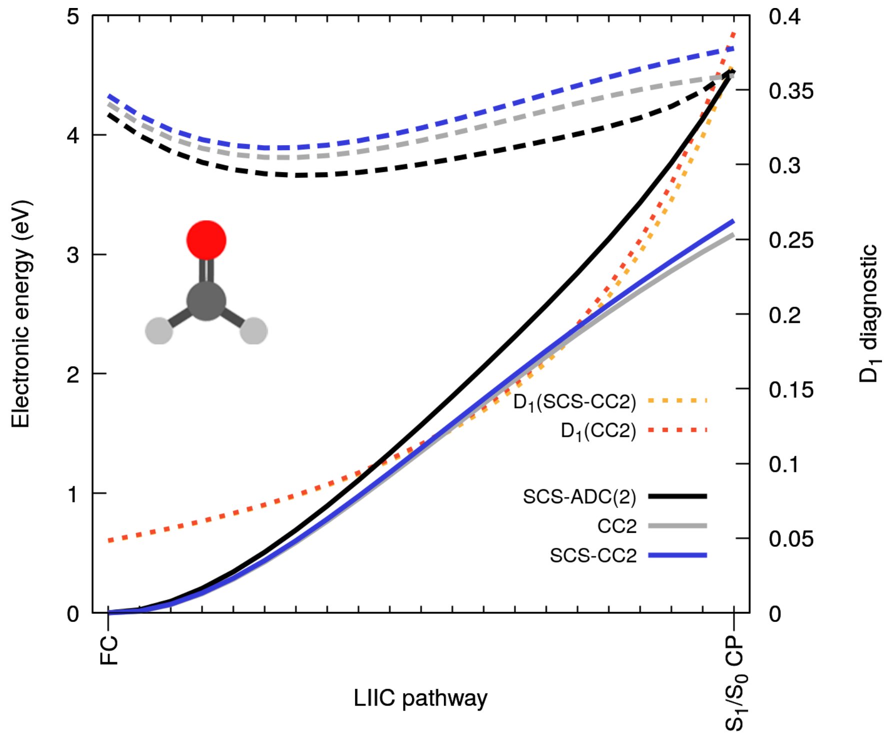

FIG. S4. Electronic energies along the LIIC pathway for formaldehyde as obtained with SCS-ADC(2)/SVP (black), CC2/SVP (grey). and SCS-CC2/SVP (blue). A solid line is used for S_0 and dashed line for S_1. The D_1 diagnostic is given by a dotted orange line (SCS-CC2) or a red line (CC2).

D. Influence of the vertical shift on XMS-CASPT2 profile

Figure S5 shows the LIIC for formaldehyde as computed with XMS(2)-CASPT2(6/5)/SVP using two shifts, 0.1 and 0.5 Hartree. Both curves are in close agreement.

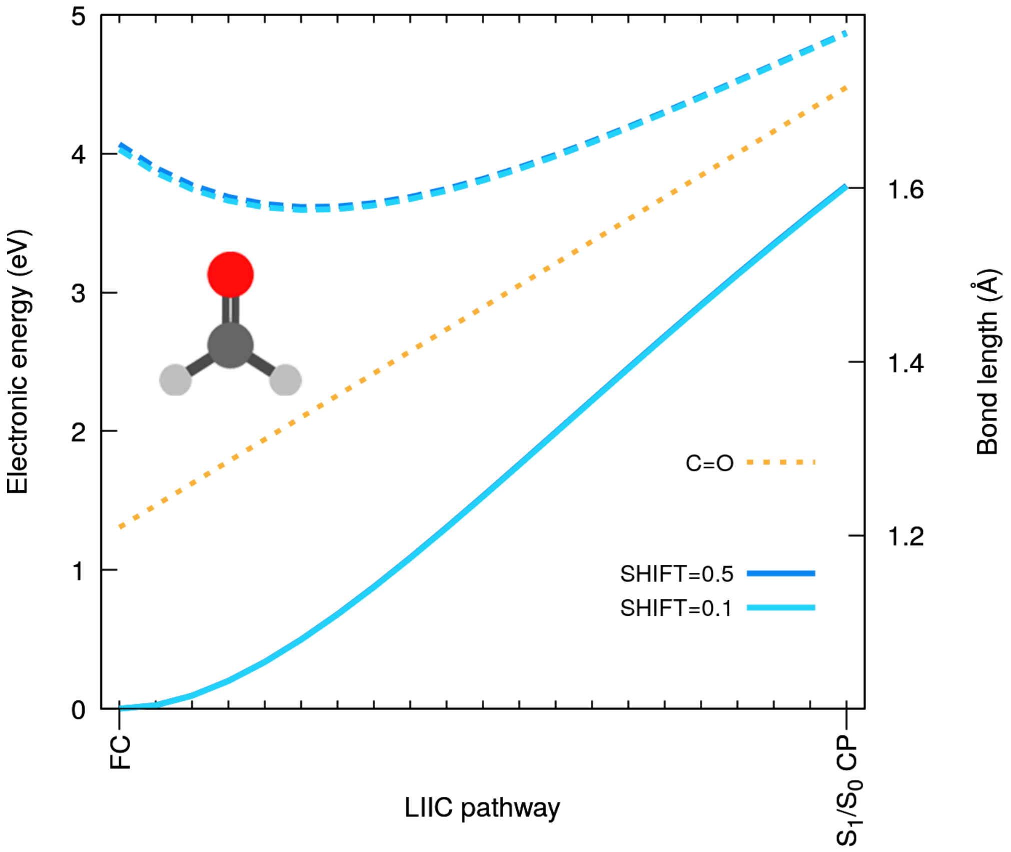

FIG. S5. Electronic energies along the LIIC pathway for formaldehyde as obtained with XMS(2)-CASPT2(6/5)/SVP using a 0.1 Ha (light blue) or 0.5 Ha (blue) shift. A solid line is used for S_0 and dashed line for S_1. The C=O bond length is given by an orange dotted line.

III. COMPARING XMS-CASPT2(2/2) WITH XMS-CASPT2(4/3) FOR ALL MOLECULES

Figure S6 reproduces the comparison (proposed in the main text for formaldehyde) between XMS-CASPT2(2/2) and XMS-CASPT2(4/3) for all the molecules discussed in this work. The XMS-CASPT2(2/2) calculations only incorporate the carbonyl n and π^* orbitals in the active space, while the XMS-CASPT2(4/3) ones include in addition its π orbital (all natural orbitals are given in Fig. S1 above). As observed for the case of formaldehyde, the (2/2) active space leads to an artificial crossing between S_1 and S_0 for all molecules (see red lines in Fig. S6), like (SCS-)ADC(2). Upon inclusion of the π orbital on the carbonyl in the active space (XMS-CASPT2(4/3), blue lines in Fig. S6), the artificial crossing is removed and the LIICs obtained are in line with their corresponding XMS-CASPT2 reference calculations.

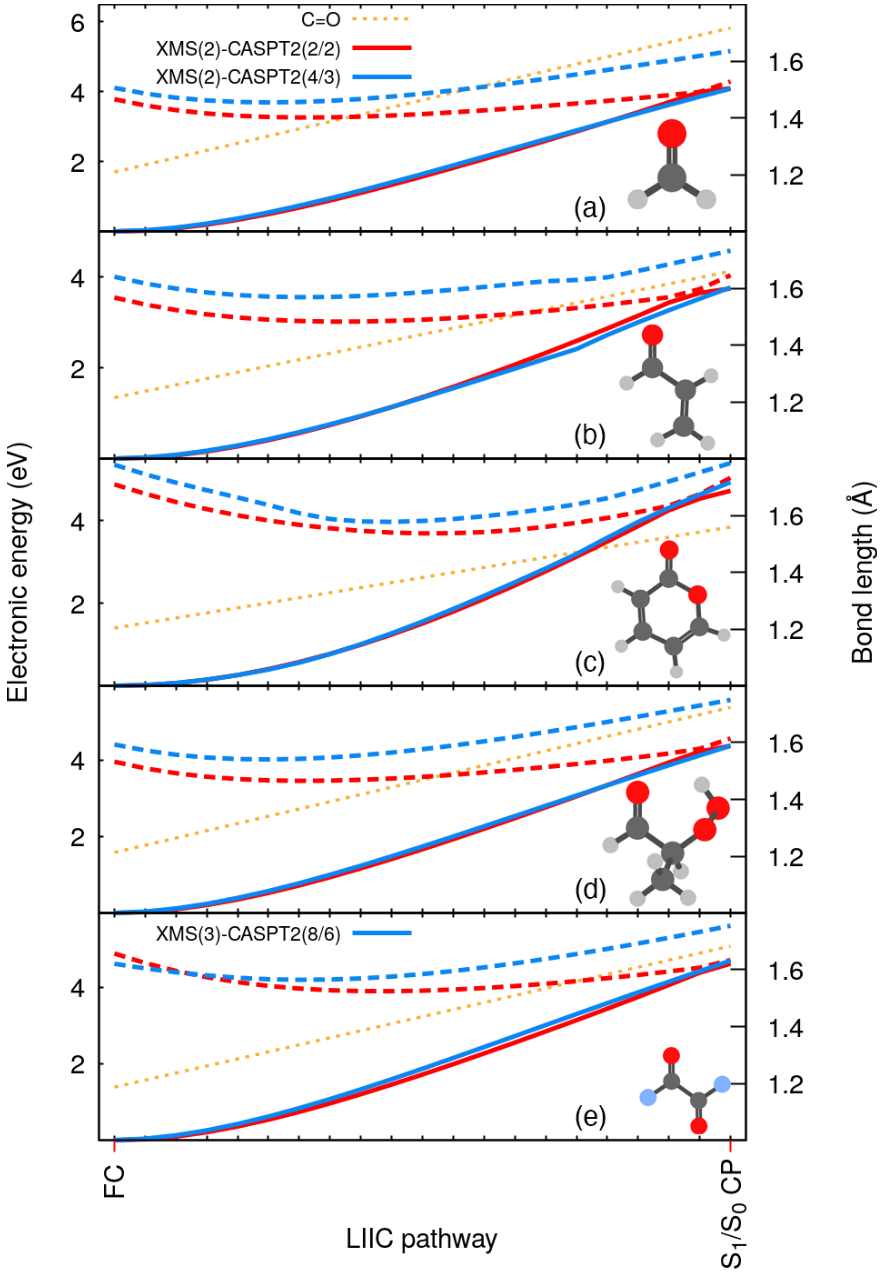

FIG. S6. Electronic energies along along the LIIC pathway for formaldehyde as obtained with XMS-CASPT2(2/2)/SVP (red) and XMS-CASPT2(4/3)/SVP (blue) for: (a) formaldehyde, (b) acrolein, (c) pyrone, (d) 2-HPP, and (e) oxalyl fluoride. We note that for oxalyl fluoride the (4/3) active space does not describe S_1 accurately and a (8/6) active space is used instead. A solid line is used for S_0 and dashed line for S_1. The C=O bond length is given by an orange dotted line.

IV. LIIC FOR THYMINE

Figure S7 presents the LIIC computed for thymine (not discussed in the main text). The profile shows that SCS-ADC(2) also exhibits a fictitious crossing between S_1 ($n\pi^*$ on one of the C=O) and S_0, in comparison with the reference provided by XMS(5)-CASPT2(12/9). We also show that XMS(2)-CASPT2(2/2) reproduces the artificial crossing, in line with the other molecules (see Fig. S6 above).

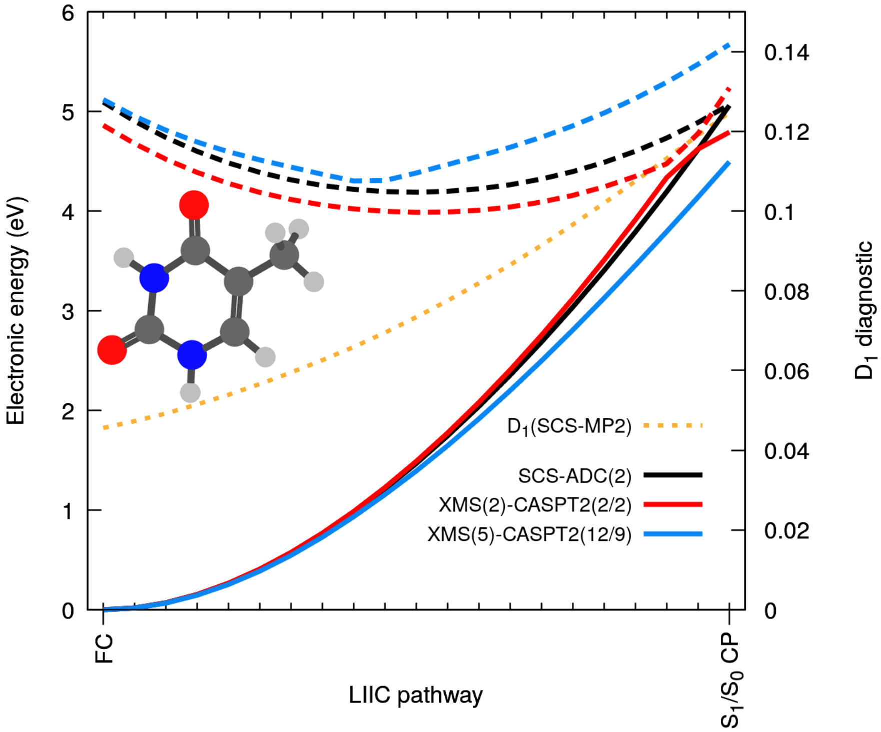

FIG. S7. Electronic energies along the LIIC pathway for thymine as obtained with SCS-ADC(2)/SVP (black), XMS(2)-CASPT2(2/2)/SVP (red) and XMS(5)-CASPT2(12/9)/SVP (blue). A solid line is used for S_0 and dashed line for S_1. The D_1 diagnostic for the SCS-MP2 ground state is shown with a dotted orange line.

REFERENCES

[1]C. Møller and M. S. Plesset, Phys. Rev. **46**, 618 (1934).

[2]C. Hättig, Advances in Quantum Chemistry **50**, 37 (2005).

[3]A. B. Trofimov and J. Schirmer, J. Phys. B: At. Mol. Opt. Phys. **28**, 2299 (1995).

[4]A. Dreuw and M. Wormit, Wiley Interdiscip. Rev. Comput. Mol. Sci. **5**, 82 (2015).

[5]O. Christiansen, H. Koch, and P. Jørgensen, Chem. Phys. Lett. **243**, 409 (1995).

[6]A. Hellweg, S. A. Grün, and C. Hättig, Phys. Chem. Chem. Phys. **10**, 4119 (2008).

[7]W. Kohn and L. J. Sham, Phys. Rev. **140**, A1133 (1965).

[8]R. G. Parr and W. Yang, *Density functional theory of atoms and molecules* (Oxford University Press, 1989).

[9]E. Runge and E. K. U. Gross, Phys. Rev. Lett. **52**, 997 (1984).

[10]M. E. Casida, in *Recent Advances In Density Functional Methods, Part I* (World Scientific, 1995) pp. 155–192.

[11]T. Helgaker, P. Jørgensen, and J. Olsen, *Molecular Electronic-Structure Theory* (John Wiley and Sons, Ltd, Chichester, UK, 2000).

[12]T. Shiozaki, W. Gyroffy, P. Celani, and H. J. Werner, J. Chem. Phys. **135** (2011), 10.1063/1.3633329.

[13]J. W. Park and T. Shiozaki, J. Chem. Theory Comput. **13**, 3676 (2017), arXiv:1706.00156.

[14]B. Vlaisavljevich and T. Shiozaki, J. Chem. Theory Comput. **12**, 3781 (2016).

[15]L. González, *Quantum Chemistry and Dynamics of Excited States: Methods and Applications* (Wiley, 2020).

[16]H. J. Werner and P. J. Knowles, J. Chem. Phys. **89**, 5803 (1988).

[17]M. Frisch, G. Trucks, H. Schlegel, G. Scuseria, M. Robb, J. Cheeseman, G. Scalmani, V. Barone, B. Mennucci, G. Petersson, *et al.*, Inc., Wallingford CT.

[18]F. Furche, R. Ahlrichs, C. Hättig, W. Klopper, M. Sierka, and F. Weigend, Wiley Interdiscip. Rev. Comput. Mol. Sci. **4**, 91 (2014).

[19]T. Shiozaki, Wiley Interdiscip. Rev. Comput. Mol. Sci. **8**, e1331 (2018).

[20]H.-J. Werner, P. J. Knowles, G. Knizia, F. R. Manby, and M. Schütz, Wiley Interdiscip. Rev. Comput. Mol. Sci. **2**, 242 (2012).

[21]H.-J. Werner, P. J. Knowles, G. Knizia, F. R. Manby, M. Schütz, P. Celani, T. Korona, R. Lindh, A. Mitrushenkov, G. Rauhut, *et al.*, See http://www. molpro. net (2012).

[22]J. P. Perdew, M. Ernzerhof, and K. Burke, J. Chem. Phys. **105**, 9982 (1996).

[23]C. Adamo and V. Barone, J. Chem. Phys. **110**, 6158 (1999).

[24]F. Weigend, A. Köhn, and C. Hättig, J. Chem. Phys. **116**, 3175 (2002).

[25]F. Weigend and R. Ahlrichs, Phys. Chem. Chem. Phys. **7**, 3297 (2005).

[26]F. Weigend, Phys. Chem. Chem. Phys. **8**, 1057 (2006).

[27]D. Rappoport and F. Furche, J. Chem. Phys. **133**, 134105 (2010).

[28]B. G. Levine, J. D. Coe, and T. J. Martínez, J. Phys. Chem. B **112**, 405 (2008).

[29]D. Tuna, D. Lefrancois, Ł. Wolański, S. Gozem, I. Schapiro, T. Andruniów, A. Dreuw, and M. Olivucci, J. Chem. Theory Comput. **11**, 5758 (2015).

[30]J. C. Tully, J. Chem. Phys. **93**, 1061 (1990).

[31]M. Barbatti, M. Ruckenbauer, F. Plasser, J. Pittner, G. Granucci, M. Persico, and H. Lischka, Wiley Interdiscip. Rev. Comput. Mol. Sci **4**, 26 (2014).

[32] M. Barbatti, G. Granucci, M. Ruckenbauer, F. Plasser, R. Crespo-Otero, J. Pittner, M. Persico, and H. Lischka, "NEWTON-X: A package for Newtonian dynamics close to the crossing seam." (2016).
[33] F. Plasser, R. Crespo-Otero, M. Pederzoli, J. Pittner, H. Lischka, and M. Barbatti, J. Chem. Theory Comput. **10**, 1395 (2014).
